# Noninvasive evaluation of hemodynamics and light scattering property during two-stage mouse cutaneous carcinogenesis based on multispectral diffuse reflectance images at isosbestic wavelengths of hemoglobin

**DOI:** 10.1117/1.JBO.24.3.031020

**Published:** 2019-01-11

**Authors:** Md. Abdul Wares, Naoki Tobita, Satoko Kawauchi, Shunichi Sato, Izumi Nishidate

**Affiliations:** a Tokyo University of Agriculture and Technology, Graduate School of Bio-Applications and Systems Engineering, Tokyo, Japan; b Ministry of Fisheries and Livestock, Government of Bangladesh, Department of Livestock Services, Dhaka, Bangladesh; c National Defense Medical College Research Institute, Division of Bio-Information and Therapeutic Systems, Tokorozawa, Saitama

**Keywords:** two-stage carcinogenesis, multispectral imaging, light scattering property, hemoglobin concentration, Monte Carlo simulation

## Abstract

We investigate a multispectral imaging method to evaluate spatiotemporal changes in both cutaneous hemoglobin concentration and light scattering parameter in mouse skin through diffuse reflectance spectroscopy using the reflectance images acquired at isosbestic wavelengths of hemoglobin (420, 450, 500, and 585 nm). In the proposed approach, Monte Carlo simulation-based empirical formulas are introduced to extract the scattering power b representing the wavelength dependence of light scattering spectrum of skin tissue, as well as the total hemoglobin concentration $C_{\mathrm{th}}$ in dermal vasculatures. The use of isosbestic wavelengths of hemoglobin enables the values of $C_{\mathrm{th}}$ and b to be estimated independently of the oxygenation of hemoglobin. Experiments using *in vivo* mice two-stage chemical carcinogenesis model are performed to confirm the feasibility of the proposed method for evaluating the changes in cutaneous vasculatures and tissue morphology during tumor initiation, promotion, and progression processes. The experimental results reveal that the changes in scattering power b of back skin are significantly reduced and followed by the increase in total hemoglobin concentration $C_{\mathrm{th}}$ in the carcinogenesis mice group, which indicates morphological changes in skin tissue such as edema and cell swelling caused by tumor promotion and successive angiogenesis along with tumor progression. The results suggest that the potential of the present method to detect cutaneous carcinogenesis in an early stage and monitor physiological changes during promotion and progression process of nonmelanoma tumors.

## 1 Introduction

Skin is the largest and most extended organ of the body and its weight goes up to about 15% of the total bodyweight.^1^ It has many functions including protecting the body against infections and the electromagnetic radiations.^2^ During its action, skin, sometimes, is affected by the heavy dose of carcinogen such as ultraviolet light from the sun, using sunlamps and sunbeds or a chemical such as 7,12-dimethylbenz[a]anthracene (DMBA) that causes cancer. Uncontrolled proliferation of skin structures, both cellular and acellular, which have no designated functions, is called skin cancer. Of all human cancerous growth, more than 90% emerges from the epithelia that are bound by the basement-membrane zone and invading of this border is the main cause of conversion of the epithelial cancers to malignancy.^3^ In this study, we focused on the nonmelanoma skin cancer (NMSC) as it is the most common type of cancer affecting mainly the light-skinned individuals.^4^ Among NMSC, basal cell carcinoma (BCC) and squamous cell carcinoma are the two most common subtypes. In fact, BCC is the most common cancer in many countries of the world^5^ accounting 75% of all NMSC.^6^

The incidence of NMSC varies widely in worldwide cases with the highest rates in Australia, >1000/100,000 person-years for the BCC, and the lowest rates in parts of Africa, <1/100,000 for BCC.^4^ Unfortunately, the rate of incidence is increasing in many countries of the world, which soared up critically in the past few decades.^4^^,^^7^^–^^10^ Putting this in consideration, the National Institute for Health and Clinical Excellence guidelines referred to the need to establish the actual nature of the epidemiology of BCC.^11^ The prevalence of skin cancer is also affected by the ethnic background and geographical location such as very low incidence was observed in the close to the equator countries such as Singapore.^12^ Previous surveys and reports suggest that the male is higher in risk than their female counterparts^13^^–^^15^ because of higher outdoor activities that results in higher exposure to the radiation and affiliation with some radiologically hazardous position. Though the mortality rate is low,^16^ NMSC causes significant economic burden to medical service and accelerate morbidity as NMSCs mostly occur in visible areas of the skin.^4^ That is why control and prevention of NMSC are highly important and are assisted by the precise diagnosis considering the forms, structure, and nature of the skin.

Structurally skin is enriched with the long cylindrical fibrous structures as well as the chromophores such as oxygenated hemoglobin, deoxygenated hemoglobin, and melanin.^1^ In case of carcinogenesis, there are significant changes in those structures those can be treated as the landmarks. The most important point to promote the recovery process or to escalate the survival of patients is the identification of inception of the disease at the earliest possible time and doing it precisely.^17^^–^^19^ In the course of carcinogenesis, the blood supply to the carcinogen-exposed area becomes increased, which is called field carcinogenesis. Following that, hyperemia starts as a result of angiogenesis relatively earlier than expression of any external changes.^20^^–^^22^ It is also characterized by the multifocal or nonhomogenous hyperplastic changes.^22^ It contributes to the hemodynamic changes. Also the skin cancer diagnostic is commonly associated to a change in the skin rigidity with respect to its surrounding area that can be checked through palpation. This rigidity change is expressed by a change in the skin’s Young’s modulus of the affected area, a feature that can be retrieved with optical nondestructive techniques.

Conventionally, carcinogenicity as well as other cutaneous disorders is diagnosed mainly based on the gross morphological parameters such as the ABCD rule^23^ and the 7-point check list.^24^ Lack of equality or equivalence between parts or aspects of the prospective part of organ or body, i.e., asymmetry, irregular border of that parts, color variation within the particular mass of the tissue, increasing the diameter in relation to time, and evolvement over time are the basic visible features. As those are not confirmatory, the equivocal lesions are recommended for the further examination so-called “gold standard” through biopsy or surgery along with the histopathological test^25^ for the confirmation.^4^ Though it is the most common practice, the pain or discomfort of the patient, chance of secondary infection, cumbersome, and time-consuming process degrades the feasibility of application. Skin carcinogenesis is characterized by the exophytic growth^26^ of the layers of the skin, which forms papilloma in most of the cases. Unfortunately, these appear at the advanced stage of the carcinogenicity when the treatment is cumbersome and most critical is recovery rate is not satisfactory. Actually, the changes in the molecular level start following the tissue faces carcinogenic agents. So early diagnosis can be done and it is mandatory for effective treatment and soon and as completely as possible recovery that brings physical and mental well-being.

There are several protocols applied to measure the skin cancer. Identifying the biomarker molecules by the biosensors is one of the important advanced measures where antigen plays a vital role but, high expense of antibody, complicated assembling of sensors, unexpected characteristics at nanoscale, and low stability in storage are the limiting factors.^27^ More recently, dermoscopy has been implemented for clinical diagnosis^28^ such as MoleMax HD,^29^ SIAscope,^30^ SolarScan,^31^ and MelaFind^32^ are important among them. But none of these targets on enhancing the contrast of structures and morphologies with low visibility in the dermoscopic image.^25^

The randomly inhomogeneously distribution of chromophores and heterogeneously fibrous structures by the collagen bundle and lamellae characterize the optical properties of skin tissues and thus they make skin tissues suitable for the application of the optical techniques.^33^ Light absorption by the chromophore such as hemoglobin, lipid, water, and light scattering by the cutaneous long cylindrical structures such as collagen fiber, reticular fiber, elastic fiber or by the other cellular or subcellular structures represent the physiological and morphological condition of the biological tissues. The primary changes in the optical properties are because of either scattering or absorption or both by the tissues.^34^ The spectral information of the absorption coefficient $\mu_{a}(\lambda )$ indicates the probability of photon absorption per unit infinitesimal path length whereas the reduced scattering coefficient $\mu_{s}^{\prime }(\lambda )$ refers to photon scattering per unit infinitesimal path length. They can be readily assessed from the reflectance spectrum by different mathematical models.^35^ The reduced scattering coefficient $\mu_{s}^{\prime }(\lambda )$ is the combination of all kinds of scattering by the cellular and subcellular structures of different sizes^36^ and is dependent on the scattering amplitude and scattering power, which reflect the geometrical properties such as scattering power density^37^ and the size of the biological molecule,^38^ respectively. Also increase or decrease in the scattering power shows the inverse relation with the scatterer size.^38^ The alternations in scattering during the carcinogenesis are thought to be result of the variation in the size of cellular and subcellular structures and engorgement of cutaneous tissue by the long cylindrical fibrous structure that is why it is necessary to monitor scattering power to assess the pathophysiological condition of the skin. Also the fluctuation in the chromophore such as hemoglobin is directly proportional to the optical absorption.

Optical protocols have been proven as more potential tool for assessing tissue condition promptly and much accurately that assists in medical diagnosis.^39^^,^^40^ It provides excellent soft-tissue contras, excellent functions, fast data acquisition, as well as low-cost applicability.^41^ Light scattering and absorption properties of the biological tissues can be evaluated by various optical techniques, such as time-resolved measurements,^42^ a frequency-domain method,^43^ optical coherence method,^44^ a pulsed photodermal radiometry method,^45^ and spatially resolved measurements.^46^^–^^49^ Diffuse reflectance spectroscopy is a promising technique to measure the macroscopic optical properties such as light scattering and absorption parameters.^50^^,^^51^ Some of the previous reports have been published on skin chromophore and morphology study based on the optical fiber probe,^51^^–^^54^ integrating sphere,^55^^,^^56^ and multispectral imaging^57^^–^^59^ for data collection.

In this study, we investigated a multispectral imaging method based on the algorithm developed previously^54^ for evaluating spatiotemporal changes in both cutaneous hemoglobin concentration and tissue morphology of mice during a two-stage chemical carcinogenesis. Multispectral diffuse reflectance images at isosbestic wavelengths of hemoglobin (420, 450, 500, and 585 nm) were acquired using a simple white light emitting diode (LED), a monochromatic charged coupled device (CCD) camera, and the narrow band optical filters. The Monte Carlo simulation (MCS)-based empirical formulas are introduced to specify the scattering power b of skin tissue as well as the concentration of total blood $C_{\mathrm{th}}$.

## 2 Principle

The diffuse reflectance spectrum R(λ) of the skin is dependent on the light scattering properties originating from the long cylindrical fibrous structures as well as some other shorter cellular structures. This scattering is dominated by both of the Rayleigh scattering from the comparatively smaller structures and the Mie scattering by the comparatively larger structures of the skin tissue. The spectrum of the reduced scattering coefficient of skin tissue $\mu_{s}^{\prime }(\lambda )$ can be approximated by the following power low function:^60^^,^^61^

$$\mu_{s}^{\prime }(\lambda )=a\lambda^{-b},$$ (1)

where λ is the wavelength of light. The coefficient a and the exponent b are called the scattering amplitude and the scattering power, which are related to geometrical properties such as scatterer density^37^ and size,^38^ respectively. An increase or decrease in scattering power b reportedly produces a decrease or increase in scatterer size, respectively.^61^ Therefore, quantifying b is useful to evaluate morphological changes such as swelling or shrinkage of cellular and subcellular structures in skin tissue. On the other hand, R(λ) depends on the concentration of melanin $C_{m}$ and that of hemoglobin $C_{\mathrm{th}}$ in epidermis and hemoglobin in the cutaneous circulation. To evaluate the scattering power b and the total hemoglobin concentration $C_{\mathrm{th}}$, the following empirical formulas derived from the results of the MCS for light transport in skin tissue are considered:^54^

$$b=\alpha_{0}+\alpha_{1}A(420)+\alpha_{2}A(450)+\alpha_{3}A(500)+\alpha_{4}A(585),$$ (2)

$$C_{\mathrm{th}}=\beta_{0}+\beta_{1}A(420)+\beta_{2}A(450)+\beta_{3}A(500)+\beta_{4}A(585).$$ (3)

In those equations, the absorbance spectrum A(λ) is calculated as

$$A(\lambda )=-\log_{10}\;R(\lambda ).$$ (4)

The coefficients $\alpha_{i}$ and $\beta_{i}$ (i=0, 1, 2, 3, and 4) in Eqs. (2) and (3) can be determined statistically through multiple regression analysis (MRA). In this MRA, b and $C_{\mathrm{th}}$ are regarded as dependent variables, and A(420), A(450), A(500), and A(585) are regarded as the independent variables. We used the MCS algorithm developed by Wang et al.,^62^ to derive the diffuse reflectance at the four isosbestic wavelengths for determining reliable values of $\alpha_{i}$ and $\beta_{i}$. The simulation model treated herein consists of epidermis and dermis, where $\mu_{a}(\lambda )$ and $\mu_{s}^{\prime }(\lambda )$ are homogeneously distributed in each volume. The absorption spectrum of epidermis $\mu_{a,e}(\lambda )$ and that of dermis $\mu_{a,d}(\lambda )$ were calculated based on the concentration of melanin $C_{m}$ and the total hemoglobin concentration $C_{\mathrm{th}}$, respectively. We assumed that the values of $\mu_{s}^{\prime }(\lambda )$ for both the epidermis and the dermis are identical. The reduced scattering spectrum $\mu_{s}^{\prime }(\lambda )$ was deduced based on the scattering amplitude a and the scattering power b. The thicknesses of the epidermis and dermis were assumed to be 0.06 and 4.94 mm, respectively. Then the spectra of $\mu_{a}(\lambda )$ and $\mu_{s}^{\prime }(\lambda )$ were given as inputs for the MCS, whereas the diffuse reflectance at 420, 450, 500, and 585 nm was derived as the outputs. The input values of $C_{\mathrm{th}}$ and b, and the resultant values of A(420), A(450), A(500), and A(585) are useful as the data set in statistically determining the values of $\alpha_{i}$ and $\beta_{i}$ for determining the absolute values of b and $C_{\mathrm{th}}$. The five different values, namely, $5.3\times 10^{9}$, $5.8\times 10^{9}$, $6.3\times 10^{9}$, $6.8\times 10^{9}$, and $7.3\times 10^{9}$, were calculated by multiplying the typical value^63^ of a by 0.8, 0.9, 1.0, 1.1, and 1.2. In the same way, the five values of 2.90, 2.95, 3.00, 3.05, and 3.10 were obtained by multiplying the typical value^64^ of b by 0.97, 0.985, 1.0, 1.015, and 1.03, respectively. Twenty-five spectra of reduced scattering coefficients $\mu_{s}^{\prime }(\lambda )$ for skin tissues were calculated using Eq. (1). The absorption coefficients of the epidermis were derived for 10 different concentrations of $C_{m}=1$ to 10 vol. % at intervals of 1%, whereas those of the dermis were derived for five different concentrations of $C_{\mathrm{th}}=0.2$ to 1.0 vol. % at intervals of 0.2 vol. %. In total, 1250 diffuse reflectance spectra were derived using every possible combination of $C_{m}$, $C_{\mathrm{th}}$, a, and b. The use of isosbestic wavelengths of hemoglobin makes it possible to eliminate the effects of the variations in hemoglobin oxygen saturation on the estimated values of $C_{\mathrm{th}}$ and b. Once we determine the empirical formulas for total hemoglobin concentration $C_{\mathrm{th}}$ and the scattering parameter b, the images of $C_{\mathrm{th}}$ and b can be reconstructed by applying the empirical formulas to each pixel of the measured spectral diffuse reflectance images, without the MCSs. In other words, the MCS is time-consuming for preparing data sets of diffuse reflectance spectra but does not significantly increase the imaging processing time for $C_{\mathrm{th}}$ and b. The uniqueness of the proposed method compared to the other spectral imaging techniques for skin measurements is the use of diffuse reflectance images acquired at the isosbestic wavelengths of hemoglobin, which make it possible to visualize spatial maps of the total hemoglobin concentration $C_{\mathrm{th}}$ and the scattering parameter b simultaneously, independent of the oxygenation.

## 3 Materials and Methods

### 3.1 Animal Preparation

In experimental cases, mice tissue serves as a productive model for human cancer and facilitates production of cancers induced both by carcinogens and defined genetic elements.^64^ Mouse skin has a faster epidermal turnover and is easier to transform using carcinogen, which is the most important mutagenic driver of skin cancer.^65^ Animal care and experimental procedures were approved by the Animal Research Committee of Tokyo University of Agriculture and Technology (Approval No. 28-50, July 13, 2016). Fifteen hairless male albino mice, 6-week old (Hos:HR-1, Hoshino Laboratory Animals Inc., Ibaraki, Japan), were housed in a controlled environment (24°C, 12-h light/dark cycle) with food and water ad libitum. Hairless mice strains proved as more effective for skin cancer study^66^ and it has no extra burden of managing hair. All mice were anesthetized and maintained with 1.0% isoflurane in the experiments. Mice were divided into three groups.

**Fig. 1:**
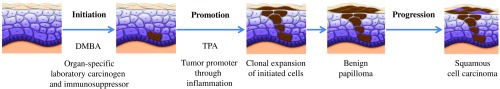
Schematic illustration of two-step carcinogenesis model induced by DMBA and TPA.

According to the cancer pathogenesis, we intend to induce two-stage chemical carcinogenesis in the back skin of mice as shown in Fig. 1. All mice were anesthetized and maintained with 1.0% isoflurane in the experiments. Mice were divided into three groups. Skin cancer was initiated with 100  μg of DMBA in 100  μl acetone in the first group of 5 mice. Mice skin shows greater percutaneous absorption and decreased barrier function^67^ so it is readily absorbed by the skin. Typically, the chemical binds with the DNA and causes mutation in *Ha-ras* gene,^68^ it is called initiation. One week later, all mice in the first group were treated with 1  μg of 12-O-tetradecanoylphorbol-13-acetate (TPA) in 100  μl of acetone was applied twice in a week for the promotion and progression of carcinogenesis for up to 26 weeks (DMBA-TPA group). TPA leads to the clonal expansion of the initiated cell that turns the initiated cells into multiple benign papillomas.^68^ Some additional genetic changes such as elevated expression of genes encoding *Ha-ras* and cyclin D1 and loss of p53 lead to conversion of benign papilloma to malignant carcinoma.^68^ This two-stage chemical carcinogenesis can cause skin tumors as early as 6 weeks with malignant conversion at 18 weeks.^69^ On the other hand, the second group of 5 mice was treated with 100  μl of acetone at first and then 1  μg of TPA in 100  μl of acetone was applied twice in a week up to 26 weeks (TPA group). The third group of 5 mice was treated with only 100  μl of acetone twice in a week up to 26 weeks (control group).

Microscopically, the epidermal thickness for DMBA-TPA group is supposed to be increased.^70^^–^^72^ The cancer cells should be increased in number^72^ and size^73^^,^^74^ and rounder in shape as cancerous cells belong lower amount of adhesive materials, such as E-cadherin, to closely bind or attach with the adjacent cells or extracellular matrix.^75^ Also the cancer cells are cytomorphologically different from the normal cell such as higher amount of nuclear materials, varied in cell size, abnormal DNA molecules as a result of mutation by the DMBA solution.^76^ TPA group should have little increase in skin thickness as a result of hyperplasia caused by the inflammation posed by the application of TPA solution.^77^ On the other hand, the control group should not have any alteration in the skin morphology.^72^

### 3.2 Imaging System

**Fig. 2:**
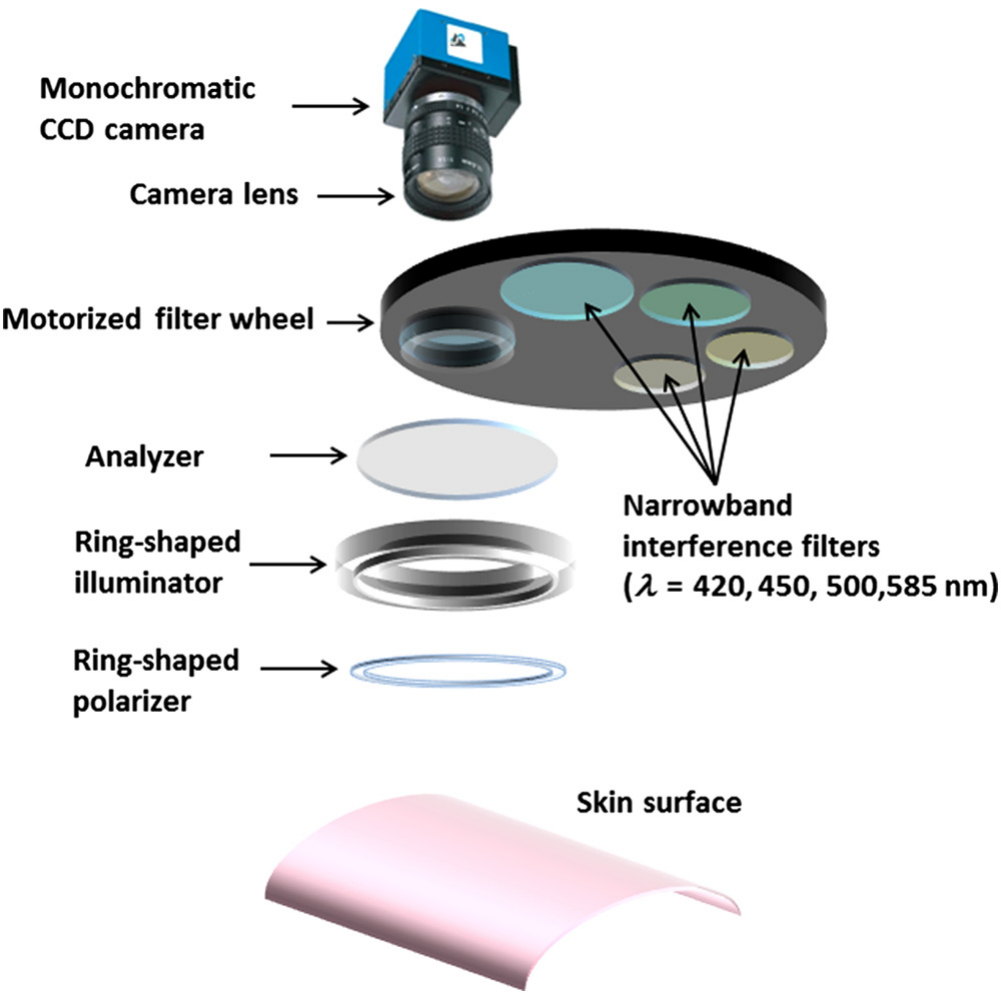
Schematic diagram of the imaging system.

To monitor progress of carcinogenesis along with changes in the total hemoglobin concentration $C_{\mathrm{th}}$ and the scattering power b, a target area on the back skin of each mouse was measured by a multispectral imaging system once in a week. Figure 2 shows a schematic diagram of the imaging system. A white-LED (LA-HDF158A, Hayashi Watch Works Co., Ltd., Tokyo, Japan) illuminated the back skin of mouse via a light guide and a ring-shaped illuminator with a primary polarization plate (polarizer). The white-LED is an inexpensive broad band light source and could be useful in clinical practice. A filter wheel was placed to filter light at isosbestic wavelengths of hemoglobin, 420, 450, 500, and 585 nm. The diffusely reflected light from the sample was captured by an 8-bit monochromatic CCD camera (DMK-21BU618.H, Imaging Source LLC, Charlotte, North Carolina) through a secondary polarization plate (analyzer) and a filter wheel installed with the four narrow band interference filters. We used the four interference filters with the central wavelength±bandwidth of 450±10  nm (F10-420.0-4-1.00, CVI laser LLC, Albuquerque, New Mexico), 450±10  nm (F10-450.0-4-25.0M, CVI Melles Griot, Albuquerque, New Mexico), 500±10  nm (F10-500.0-4-25.0M, CVI Melles Griot, Albuquerque, New Mexico), and 585±10  nm (F10-585.0-4-1.00, CVI laser LLC, Albuquerque, New Mexico). The image size of the camera was 640×480  pixels. The polarizer and the analyzer were placed in a crossed-Nicols alignment to reduce specular reflection from the skin surface. The spectrum of diffusely reflected light intensity from a standard white diffuser (SRS-99-020, Labsphere Incorporated, North Sutton, New Hampshire) $I_{w}(\lambda )$ was used to normalize the spectrum of diffusely reflected light intensity from the skin tissue $I_{s}(\lambda )$. The normalized spectrum was treated as the diffuse reflectance spectrum of the skin tissue R(λ). The diffuse reflectance images were stored in a personal computer and analyzed. The images of scattering power b and total hemoglobin concentration $C_{\mathrm{th}}$ were estimated by applying the empirical formulas described in Sec. 2 to each pixel of the diffuse reflectance images at 420, 450, 500, and 585 nm.

To monitor the changes in total hemoglobin concentration $C_{\mathrm{th}}$ and scattering power b induced by application of DMBA, TPA, and/or acetone, the relative changes in $C_{\mathrm{th}}$ and b were calculated based on the time course data. The values of $C_{\mathrm{th}}$ and b before treating with the chemical agents were used as control values, $C_{\mathrm{th},c}$ and $b_{c}$, respectively. The value of $C_{\mathrm{th},c}$ or $b_{c}$ was subtracted from each of the subsequent value of $C_{\mathrm{th}}$ or b in the time course, respectively. Each subtracted value demonstrated the change in $C_{\mathrm{th}}$ or b, $C_{\mathrm{th}}-C_{\mathrm{th},c}$ or $b-b_{c}$, over time, was normalized by dividing $C_{\mathrm{th}}-C_{\mathrm{th},c}$ by $C_{\mathrm{th},c}$, or $b-b_{c}$ by $b_{c}$. The relative changes in $C_{\mathrm{th}}$ and b were expressed as $\Delta C_{\mathrm{th}}\%=[(C_{\mathrm{th}}-C_{\mathrm{th},c})/C_{\mathrm{th},c}]\times 100$ and $\Delta b\%=[(b-b_{c})/b_{c}]\times 100$. Those calculations were applied to the time courses of $C_{\mathrm{th}}$ and b for all mice in each group.

### 3.3 Phantom Experiment

To confirm the validity of the method, we performed experiments using an agarose-based phantom that mimics the optical properties of biological tissue. The phantom consists of an epidermis layer and a dermis layer. Detailed explanations of the protocol for making the phantom have been described elsewhere.^78^ We prepared agar solution by diluting agarose powder (Fast Gene AG01, NIPPON Genetics EUROPE GmbH, Düren, NRW, Germany) with saline at a weight ratio of 1.0%. To simulate the scattering condition, Intralipid 10% solution (Fresenius Kabi AB, Uppsala, Sweden) was added to the agarose solution. The resultant solution was used as the base material. The volume concentration of Intralipid 10% solution ranged from 10% to 15%. An epidermis phantom layer was made by adding a coffee solution to the base materials. The volume concentration of coffee solution was 5%. A dermis phantom layer was made by adding a small amount of fully oxygenated horse blood with Hct=44% to the base material. All phantoms were hardened in molds having the required thickness and size by being cooled at 5°C for 20 min. These phantom layers were then piled to be a two-layered phantom, which was put between two slide glasses. The slide glass on the epidermis layer was naturally coupled to the phantom by a drop of saline. We made 12 optical phantoms with different combinations of $C_{\mathrm{th}}$ and b.

As preparatory measurements, we first determined the absorption coefficients $\mu_{a}$ and reduced scattering coefficient of $\mu_{s}^{\prime }$ each phantom at 420, 450, 500, and 585 nm, because they are necessary for the MCS to deduce the empirical formula for $C_{\mathrm{th}}$ and b. The spectra of the absorption coefficient $\mu_{a}(\lambda )$ and reduced scattering coefficients $\mu_{s}^{\prime }(\lambda )$ are also required to calculate the given values for $C_{\mathrm{th}}$ and b. For this purpose, we measured the diffuse reflectance and total transmittance spectra of each phantom individually. A 150-W halogen-lamp light source (LA-150SAE, Hayashi Watch Works Co., Ltd., Tokyo, Japan) illuminated the phantom via a light guide (LGC1-5L1000; Hayashi Watch Works Co., Ltd., Tokyo, Japan) and lens with a spot diameter of 2.0 mm. The diameter and focal length of the lens are 50 and 100 mm, respectively. The thickness of each phantom in the preparatory measurements was 1.0 mm, whereas the area of each phantom was $26\times 45\;{\mathrm{mm}}^{2}$. The phantom was placed between two glass slides having a thickness of 1.0 mm and fixed at the sample holder of an integrating sphere (RT-060-SF, Labsphere Incorporated, North Sutton, New Hampshire). The detected area of the phantom was circular with a diameter of 22 mm. Light diffusely reflected from the detected area was received at the input face of an optical fiber probe having a diameter of 400  μm, which was placed at the detector port of the sphere. The fiber transmits the received light into a multichannel spectrometer (USB2000, Ocean Optics Inc., Dunedin, Florida), which measured reflectance or transmittance spectra in the visible to near-infrared wavelength region under the control of a personal computer. To determine $\mu_{a}(\lambda )$ and $\mu_{s}^{\prime }(\lambda )$ from the measured diffuse reflectance and total transmittance spectra, we utilized the inverse Monte Carlo (IMC) method.^78^ In the IMC, the MCS of the reflectance and transmittance spectra were iterated for different values of $\mu_{a}(\lambda )$ and $\mu_{s}^{\prime }(\lambda )$ until the difference between the simulated and measured spectral values decreased below a predetermined threshold. The values used in the last step of the iteration were adopted as the final results. This process was carried out at 420, 450, 500, and 585 nm, and wavelength-dependent properties of $\mu_{a}(\lambda )$ and $\mu_{s}^{\prime }(\lambda )$ were obtained for each phantom. In these calculations, the refractive index was assumed to be 1.33 for all phantoms in the whole wavelength range. The concentration of total hemoglobin $C_{\mathrm{th}}$ in each phantom was calculated based on the estimated $\mu_{a}$ at 585 nm and the known value of extinction coefficient of hemoglobin. The scattering parameter b for each phantom was calculated from the estimated $\mu_{s}^{\prime }(\lambda )$ based on Eq. (1). Those values of $C_{\mathrm{th}}$ and b obtained by IMC are used as the given values to evaluate the validity of the proposed method experimentally. We also need to have the empirical formulas for the phantoms used in this study. We generated diffuse reflectance spectra at 420, 450, 500, and 585 nm using the MCS with the conditions of the phantoms. For this simulation, the values of $\mu_{a}(\lambda )$ and $\mu_{s}^{\prime }(\lambda )$ at 420, 450, 500, and 585 nm were set to be the same as those estimated by IMC. We derived the empirical formulas for estimating $C_{\mathrm{th}}$ and b.

## 4 Results and Discussion

**Fig. 3:**
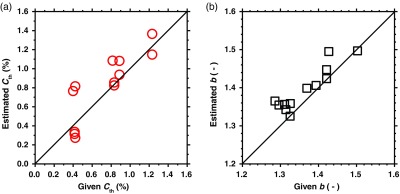
Comparisons between the estimated and given values for: (a) total hemoglobin $C_{\mathrm{th}}$ and (b) scattering parameter b, obtained from the phantom experiments.

Figure 3 shows the comparisons between the estimated and given values for (a) total hemoglobin $C_{\mathrm{th}}$ and (b) scattering parameter b, obtained from the phantom experiments. In Figs. 3(a) and 3(b), the estimated values are well correlated with the given values. Correlation coefficients between the estimated and given values are 0.85 (P<0.001) and 0.92 (P<0.0001) for $C_{\mathrm{th}}$ and b, respectively. These results indicate the validity of the proposed method for estimating the total hemoglobin $C_{\mathrm{th}}$ and scattering parameter b.

**Fig. 4:**
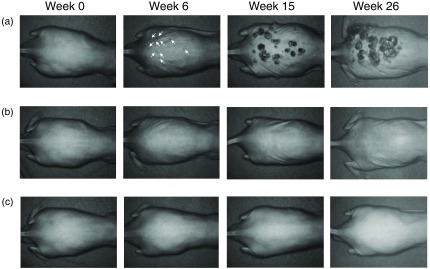
Typical results of reflected light intensity images at 450 nm obtained from: (a) DMBA-TPA group, (b) TPA group, and (c) control group at the specific time points.

Figure 4 shows the typical results of reflected light intensity images at 450 nm obtained from (a) DMBA-TPA group, (b) TPA group, and (c) control group at the specific time points. There were no remarkable changes in reflected light intensity and textural pattern in the images of skin for control group as shown in Fig. 4(c). On the other hand, several small dark spots [white arrows in Fig. 4(a)] can be observed on the back skin area in the image obtained from DMBA-TPA group at sixth week. Reductions in reflected light intensity around the dark spots imply an increase in absorption of light by hemoglobin due to angiogenesis in tumor growth or a decrease in a scattering intensity of light due to changes in tissue morphology.

In the next step, we visualized two parameters, the total hemoglobin concentration $C_{\mathrm{th}}$ and the scattering power b for assessing hemodynamic changes in cutaneous vasculatures and morphological changes in skin tissue under the specific experimental conditions, respectively. Actually, it might be related to the fact that a specific tissue condition reflects the particular pathologies.^69^

**Fig. 5:**
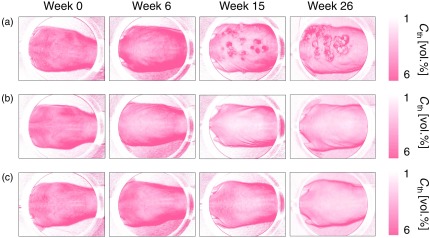
Typical resultant images of total hemoglobin concentration $C_{\mathrm{th}}$ obtained from: (a) DMBA-TPA group, (b) TPA group, and (c) control group at the specific time points.

**Fig. 6:**
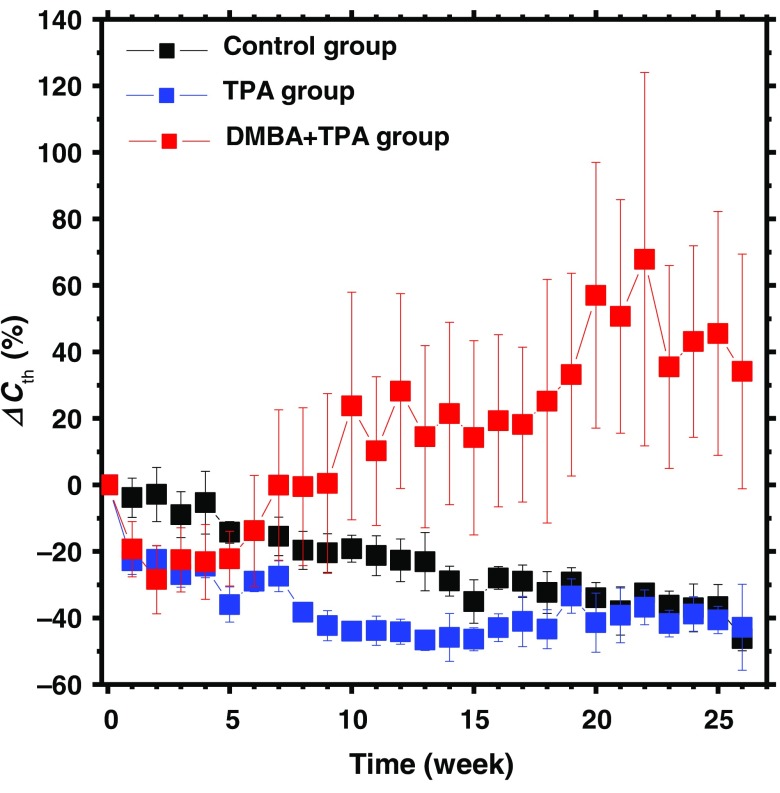
Time courses of relative change in total hemoglobin concentration $\Delta C_{\mathrm{th}}$ averaged over the five ROIs for all five mice in each group.

Figure 5 shows the typical resultant images of total hemoglobin concentration $C_{\mathrm{th}}$ obtained from (a) DMBA-TPA group, (b) TPA group, and (c) control group at the specific time points. Figure 6 shows the time courses of relative change in total hemoglobin concentration $\Delta C_{\mathrm{th}}$ averaged over the five regions of interest (ROIs) for all five mice in each group. The ROIs for DMBA-TPA group covered roughly the same dark spot regions in each mouse over time. The ROIs for TPA group and control group were randomly selected and covered roughly the same skin regions in each mouse. The value of $\Delta C_{\mathrm{th}}$ for DMBA-TPA group was started to increase on sixth week, which indicates angiogenesis causing enrichment of skin with enormous supply of blood vessels that lodge blood. Lowering of $\Delta C_{\mathrm{th}}$ for both control group and TPA group might be the fact that the effect of light absorption by hemoglobin in dermal vasculatures on the diffuse reflectance spectrum becomes lower, accompanied with skin thickening over the period as mice growing older.

We found pinkish discoloration of the target area of the skin in the initial days of the cancer induction that gradually turned into the reddish, which is prominently found in the exophytic growth area called papilloma. This is supposed to be skin carcinogenesis. The findings are backed up by some other previous researches with different protocols.^25^^,^^26^^,^^79^^–^^81^ At the advanced stage, we found the blackish discoloration of the skin in the tip of the papilloma when it experienced necrosis. There is a report that describes the measurement of melanin content as a diagnostic tool for cutaneous melanoma.^82^ We recommend extending the measurement up to the study of a light scattering property in addition to the evaluation of total hemoglobin concentration.

**Fig. 7:**
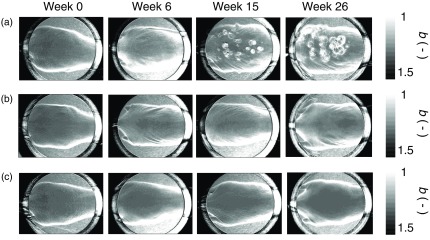
Typical resultant images of scattering power b obtained from: (a) DMBA-TPA group, (b) TPA group, and (c) control group at the specific time points.

**Fig. 8:**
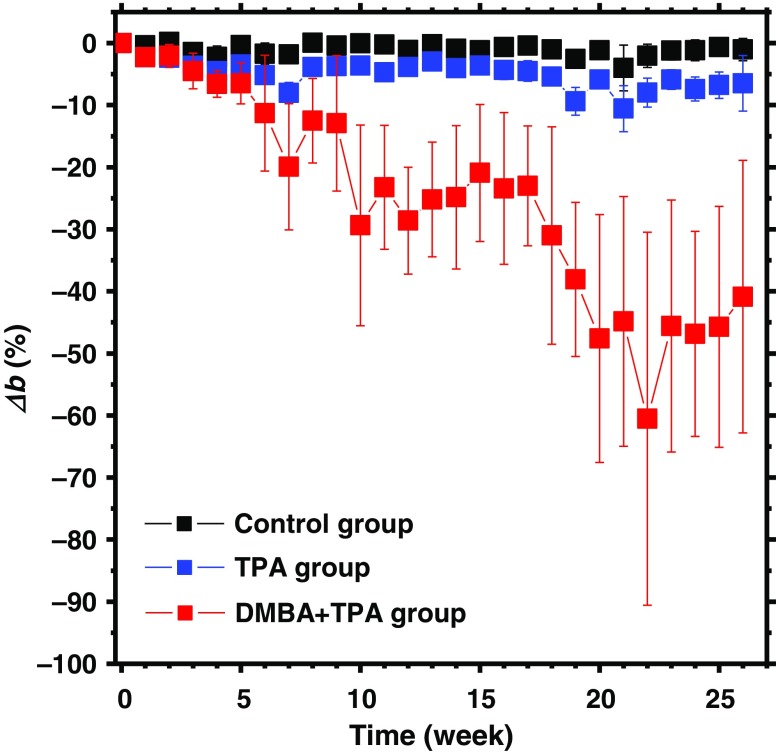
Time courses of relative change in scattering power Δb averaged over the five ROIs for all five mice in each group.

Figure 7 shows the typical resultant images of scattering power b obtained from (a) DMBA-TPA group, (b) TPA group, and (c) control group at the specific time points. Figure 8 shows the time courses of relative change in scattering power Δb averaged over the five ROIs for all five mice in each group. There was no significant change in Δb for control group over time. TPA group showed lower value of Δb than control group, which is indicative of a slight increase in skin thickness as a result of hyperplasia caused by the repeated inflammatory reactions to the TPA applications^77^ as described in Sec. 3.1. On the other hand, the value of Δb for DMBA-TPA group was started to decrease rapidly on the fifth week of the experiment, which implies the increase in size of cancer cells^73^^,^^74^ as described in Sec. 3.1. Interestingly, the rapid drop in Δb is one to two week earlier than the significant increase in $\Delta C_{\mathrm{th}}$, which might be due to the engorgement of skin with long, cylindrical fibrous structures such as collagen fiber, reticular fiber, and elastic fiber, indicating the potential of light scattering to detect cutaneous carcinogenesis in an early stage. The significant decrease in Δb observed after the seventh week indicates morphological changes in skin tissue such as edema and cell swelling caused by promotion of tumors. Macroscopically, by means of palpation, we found that the thickness and rigidity of the skin has significantly increased. This cutaneous hyperplasia further confirms the outcomes of previous research.^70^^–^^72^ The results by palpation and the proposed optical approach are consistent with each other and supported by the other research, in which cutaneous carcinogenesis increases higher concentration of densely packed collagen fibers.^65^ The use of both scattering power b and total hemoglobin concentration may be useful for earlier and more reliable diagnosis of skin cancers. The results of light scattering change and dermal hemodynamics indicate the potential of the present method to monitor nonmelanoma skin cancer promotion and progression.

**Fig. 9:**
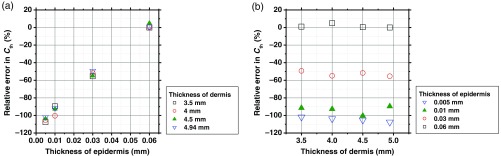
Plots of relative errors with respect to the given value of $C_{\mathrm{th}}$ for four different thicknesses of: (a) epidermis and (b) dermis.

**Fig. 10:**
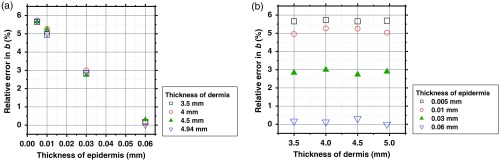
Plots of relative errors with respect to the given value of b for four different thicknesses of: (a) epidermis and (b) dermis.

We assumed that the thicknesses for the epidermis and dermis are 0.06 and 4.94 mm, respectively, in the MCS model. Changes in epidermis and dermis will affect the regression model. To investigate the effect of variation in the thicknesses of the epidermis and dermis on the estimated values of $C_{\mathrm{th}}$ and b, we performed numerical estimation for diffuse reflectance samples generated by the MCS when the thicknesses of the epidermis and dermis have some variations. For test samples, the thicknesses of epidermis were set to be 0.005, 0.01, 0.03, and 0.06 mm, whereas those of dermis were set to be 3.5, 4, 4.5, and 4.94 mm. The values of $C_{m}$, $C_{\mathrm{th}}$, a, and b were set with 1.0 vol. %, 0.4 vol. %, $6.3\times 10^{9}\;{\mathrm{cm}}^{-1}$, and 3.00, respectively. Figures 9(a) and 9(b) show the relative errors with respect to the given value of $C_{\mathrm{th}}$ for four different thicknesses of epidermis and those for dermis, respectively. The relative error in the estimated $C_{\mathrm{th}}$ decreases as the thickness of epidermis decreases, while there is no significant coupling of the estimated $C_{\mathrm{th}}$ and the thickness of dermis. Absorption of light by melanin in epidermis decreases as the thickness of epidermis is decreased. This will cause the decrease in baseline of A(λ) of skin tissue. As a consequence, the total hemoglobin concentration will be underestimated. Figures 10(a) and 10(b) show the relative errors with respect to the given value of b for four different thicknesses of epidermis and those for dermis, respectively. The relative error in the estimated b increases as the thickness of epidermis decreases, while there is no significant dependence of the estimated b on the thickness of dermis. The estimated value of b is less sensitive to the variation in epidermis thickness than that of $C_{\mathrm{th}}$. This is probably due to that the scattering parameter b represents the wavelength dependence of scattering spectrum and suffers little influence of baseline drift in A(λ) of skin tissue.

The dorsal skin surface of mouse is obviously not very flat as shown in Fig. 4. It is easy to let undesired shading into the diffuse reflectance images through the imaging system. This nonuniformity can degrade the resultant images. The total hemoglobin concentration $C_{\mathrm{th}}$ and the scattering parameter b were overestimated due to the shading as shown in Figs. 5 and 7. The errors in $C_{\mathrm{th}}$ and b due to the shading might be reduced using the ratios A(420)/A(450), A(420)/A(500), and A(420)/A(585) as the variables in Eqs. (2) and (3). This issue should be performed in future works.

In the late stage, the tumors are big and spherical. Therefore, the tumors and the surrounding skin are not at the same distance from the imaging system. This causes the variations in the depth of field and the working distance between tumors and the surrounding skin. When the imaging is focused on the surrounding skin surface, the spatial resolutions and the accuracies for both images of total hemoglobin concentration and scattering parameter could be degraded. We used the four narrow band interference filters with the center wavelengths of 420, 450, 500, and 585 nm to acquire the diffuse reflectance images at the isosbestic wavelength of hemoglobin. Therefore, the bandwidths for the four interference filters can affect the accuracies of total hemoglobin concentration and scattering parameter.

In this study, the measurements of multispectral diffuse reflectance images for all mice in each group were conducted once in a week. Measurements at shorter time intervals should be performed to evaluate more detailed changes in b and $C_{\mathrm{th}}$ in early stage of carcinogenesis. We exclude the effect of variation in hemoglobin oxygen saturation on the estimated values of b and $C_{\mathrm{th}}$ using isosbestic wavelengths of hemoglobin. On the other hand, abnormal vasculatures and functions in tumor tissue lead to impaired oxygen delivery compared with normal tissue. Moreover, oxygen consumption by tumor tissue is increased by a high tissue metabolic rate due to tumor cell proliferation. As a consequence, oxygen consumption increases by tumor tissue. The decreased oxygen supply and increased oxygen demand keep tumor tissue in hypoxic condition. Therefore, both angiogenesis and hypoxia are critical for tumor study. In this study, we mainly targeted hemoglobin and scattering qualification to evaluate on angiogenesis and carcinogenesis. The proposed method could be expanded for oxygen saturation imaging by adding the diffuse reflectance image acquired at the other wavelengths than isosbestic wavelengths of hemoglobin into the analysis. The diffuse reflectance image at 560 nm can be potentially used for this purpose.

## 5 Conclusion

In summary, a method for imaging light scattering parameter and total hemoglobin concentration of *in vivo* skin tissue based on a set of diffuse reflectance images acquired at the four isosbestic wavelengths of hemoglobin (420, 450, 500, and 585 nm) was demonstrated in this study. *In vivo* experiments using mice two-stage chemical carcinogenesis model confirmed the feasibility of the proposed imaging method for evaluating the changes in cutaneous vasculatures and tissue morphology. The results from this study indicate the potential of the present method to monitor the changes in physiology and tissue morphology in skin tissue in nonmelanoma skin cancer promotion and progression. To the best of my knowledge, this is the first example of long-term observation of changes in light scattering parameter and total hemoglobin concentration during two-stage cutaneous carcinogenesis of *in vivo* mouse skin using a multispectral imaging technique. The results of animal experiments shown in this study have novelty and significance in the field of biomedical optics. The present algorithm can consider the empirical formulas for not only the scattering power b and total hemoglobin concentration $C_{\mathrm{th}}$ but also the melanin concentration.^54^ We intend to extend the imaging method to evaluate melanoma skin cancer in future works.
